# K_ATP_ channel mutations in congenital hyperinsulinism: Progress and challenges towards mechanism-based therapies

**DOI:** 10.3389/fendo.2023.1161117

**Published:** 2023-03-28

**Authors:** Assmaa ElSheikh, Show-Ling Shyng

**Affiliations:** ^1^ Department of Chemical Physiology and Biochemistry, Oregon Health & Science University, Portland, OR, United States; ^2^ Department of Medical Biochemistry, Tanta University, Tanta, Egypt

**Keywords:** ATP-sensitive potassium channel, insulin secretion, sulphonylurea receptor 1 (SUR1), kir6.2, hypoglycemia

## Abstract

Congenital hyperinsulinism (CHI) is the most common cause of persistent hypoglycemia in infancy/childhood and is a serious condition associated with severe recurrent attacks of hypoglycemia due to dysregulated insulin secretion. Timely diagnosis and effective treatment are crucial to prevent severe hypoglycemia that may lead to life-long neurological complications. In pancreatic β-cells, adenosine triphosphate (ATP)-sensitive K^+^ (K_ATP_) channels are a central regulator of insulin secretion vital for glucose homeostasis. Genetic defects that lead to loss of expression or function of K_ATP_ channels are the most common cause of HI (K_ATP_-HI). Much progress has been made in our understanding of the molecular genetics and pathophysiology of K_ATP_-HI in the past decades; however, treatment remains challenging, in particular for patients with diffuse disease who do not respond to the K_ATP_ channel activator diazoxide. In this review, we discuss current approaches and limitations on the diagnosis and treatment of K_ATP_-HI, and offer perspectives on alternative therapeutic strategies.

## Introduction

Congenital hyperinsulinism (CHI) is a group of clinically, genetically, and morphologically heterogeneous disorders characterized by recurrent episodes of hyperinsulinemia and hypoglycemia due to dysregulated insulin secretion from pancreatic β-cells (1, 2). This condition can lead to neonatal seizures, developmental delay, and irreversible brain damage if not promptly diagnosed and treated (3). The age of clinical presentation in general correlates with the severity of the disease (4). Severe cases show symptoms of hypoglycemia early in the neonatal life, while milder forms are usually diagnosed later in infancy or childhood with recurrent attacks of hypoglycemia, which manifest following prolonged fasting or other health stress. CHI was first named as “idiopathic hypoglycemia of infancy” (5), which is no longer used after many genetic causes of the disease have been identified (6, 7). It was also once referred to as “nesidioblastosis” based on an early suggestion that the increased insulin secretion is secondary to budding of pancreatic islets observed in histological samples from patients with CHI (8). The use of this name to describe CHI was discontinued after nesidioblastosis was revealed to be a normal fetal and neonatal phenomenon (9, 10). Another discontinued historical term is persistent hyperinsulinemic hypoglycemia of infancy or PHHI, as it is now understood that the disease can be neonatal, infantile or childhood and can persist to adulthood (11). In addition to prototypical CHI, hyperinsulinism can be a pathology of a syndromic disease, including Beckwith-Wiedemann syndrome, Perlman syndrome, Kabuki syndrome, Turner syndrome, Sotos syndrome, and others (7, 12).

The estimated incidence of CHI is 1:28,000–1:50,000 in Western populations but as high as 1:2,500 in populations with higher rates of consanguinity (3, 13, 14). Variants in at least ten genes have now been linked to congenital hyperinsulinism, including genes that encode the K_ATP_ channel subunits (*ABCC8* and *KCNJ11*), glucokinase (*GCK*), glutamate dehydrogenase (*GLUD1*), the mitochondrial enzyme 3-hydroxyacyl-CoA dehydrogenase (*HADH*), proton-linked monocarboxylate transporter (*SLC16A1*), mitochondrial uncoupling protein 2 (*UCP2*), hepatocyte nuclear factor 1 alpha (*HNF1A*) and 4 alpha (*HNF4A*), and hexokinase 1 (*HK-1*) (15). Defects in these proteins result in dysregulation of insulin secretion and impaired glucose homeostasis. Of the CHI-associated gene mutations, those in *ABCC8* or *KCNJ11* that lead to loss-of-function of K_ATP_ channels are the most common (16). The majority of K_ATP_ gene mutations identified to date are in *ABCC8*, which is much larger than *KCNJ11* (16). This review summarizes current approaches and limitations on the diagnosis and treatment of K_ATP_-HI, and offers perspectives on alternative therapeutic strategies. Readers interested in K_ATP_ channel structure-function and pharmacology are referred to several recent reviews (17–19).

## K_ATP_-HI: Molecular diagnosis

**Figure 1:**
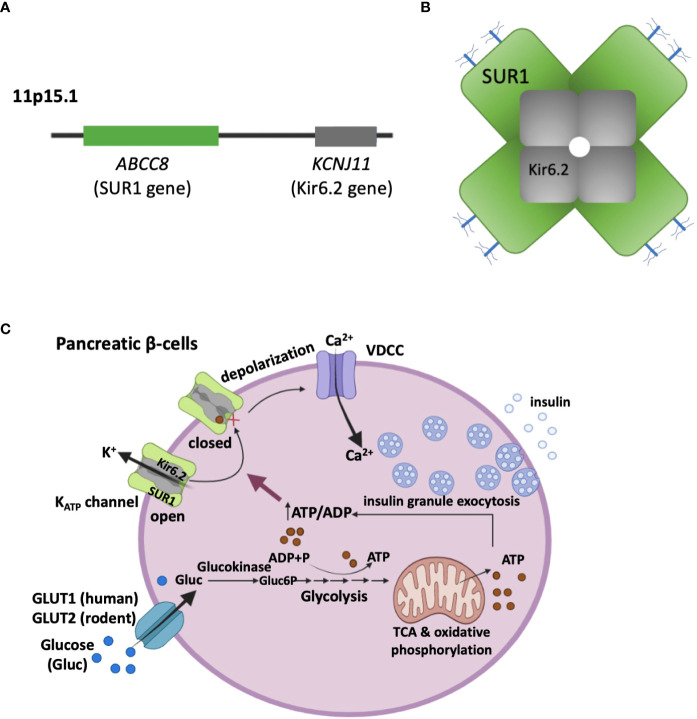
K_ATP_ channel composition and its role in coupling glucose metabolism to insulin secretion. **(A)** *ABCC8* and *KCNJ11* encoding the two pancreatic K_ATP_ channel subunits, SUR1 and Kir6.2 respectively, are located on the short arm of chromosome 11 (11p15.1). **(B)** Schematic representation of the K_ATP_ channel complex viewed in cross section. The K^+^-conducting pore is formed by four Kir6.2 subunits, which are surrounded by four SUR1 regulatory subunits. The branched blue sticks represent the two N-linked glycosylation sites in each SUR1. **(C)** β-cell K_ATP_ channels couple glucose metabolism to insulin secretion by regulating plasma membrane potential in response to varying blood glucose levels. With high serum blood glucose levels, glucose enters β-cells through glucose transporters (GLUT1 in human, and GLUT2 in rodents). Inside the cell, glucose is catabolized in the cytosol (Glycolysis) and the mitochondria (Tricarboxylic acid cycle, TCA), leading to an elevation of the ATP/ADP ratio. This results in closure of K_ATP_ channels, plasma membrane depolarization, opening of voltage-dependent Ca^2+^ channels (VDCC). The ensuing Ca^2+^ influx then triggers exocytosis of insulin secretory granules.

K_ATP_ channels have a central role in regulating insulin secretion from pancreatic β-cells in the islets of Langerhans (20, 21). The channel is composed of four pore-forming subunits Kir6.2, encoded by *KCNJ11*, and four regulatory subunits called sulfonylurea receptor 1 (SUR1), encoded by *ABCC8* (22, 23) (**Figures 1A, B**). SUR1 is so named because it binds sulfonylurea drugs, which inhibit K_ATP_ channel activity and are commonly used to treat type 2 diabetes (24). Pancreatic K_ATP_ channels are gated physiologically by intracellular ATP and ADP; ATP acts on Kir6.2 to close the channel, while MgADP acts on SUR1 to open the channel (17). This enables K_ATP_ channels to serve as metabolic sensors, coupling serum glucose to insulin secretion. At basal glucose levels, the ATP/ADP ratios are relatively low to allow K^+^ conductance through K_ATP_ channels, which sets the plasma membrane in a hyperpolarized state to prevent insulin secretion. When blood glucose levels rise, glucose metabolism increases the ATP/ADP ratio, which favors closure of K_ATP_ channels, resulting in cell membrane depolarization, activation of voltage-gated Ca^2+^ channels, and exocytosis of insulin granules (25) (**Figure 1C**). The ability of K_ATP_ channels to control β-cell membrane excitability in response to blood glucose levels is essential for glucose homeostasis. In CHI, faulty K_ATP_ channel genes that reduce or abolish functional channels in the β-cell membranes uncouple blood glucose from insulin secretion, leading to inappropriate insulin secretion despite life-threatening hypoglycemia (21, 26, 27).

Molecular diagnosis of K_ATP_-HI begins with genetic testing. Genomic DNA mutation screening of probands is done using genome isolated from peripheral blood or saliva (7, 28). However, interpreting the effect of novel *ABCC8* or *KCNJ11* variants can be challenging as they can be dominant or recessive functional mutations, or benign polymorphisms (16, 29). Recent advances combining genetic, clinical, and *in vitro* biochemical studies to determine which of the genetic variations affect transcription/splicing, translation, and function have significantly improved the diagnosis of K_ATP_-HI (30, 31).

### Focal versus diffuse K_ATP_-HI

Histologically, there exist two forms of K_ATP_-HI: focal and diffuse (15, 32). In focal disease, the defect is limited to a focal lesion in the pancreas due to a heterozygous, paternally inherited *ABCC8* or *KCNJ11* mutation coupled with loss of the normal maternal allele in a subset of pancreatic β-cells during embryonic development (28). In diffuse K_ATP_-HI the entire pancreas is affected. Patients carrying homozygous, heterozygous mutations, or rare compound heterozygous mutations have all been reported (29). The two forms of K_ATP_-HI are not easily distinguishable by clinical presentations (15). Genetic information from the proband and parents can point to possible focal disease. Indeed, in patients whose genetic testing identifies one paternally inherited recessive mutation in *ABCC8* or *KCNJ11* and no maternal mutation, there is > 95% likelihood of a focal disease (29). This can be directly confirmed by PET imaging using ^18^F-fluoro-L-DOPA. Localization of focal lesions by ^18^F-fluoro-L-DOPA PET imaging along with CT-angiography allows for surgical removal of focal lesions in most cases for a complete cure (33). By contrast, the diffuse form, if not responsive to pharmacological treatment or glucose infusion, may require near total pancreatectomy to manage hypoglycemia, leading to future complications (3, 34).

### Dominant versus recessive K_ATP_-HI

Determining whether the mutation is dominant or recessive is of great clinical importance, especially for guiding decisions on whether to conduct ^18^F-fluoro-L-DOPA PET scan to test for focal disease. Moreover, the information is important for genetic counseling concerning recurrence risk as well as for identifying other family members at risk for hypoglycemia (35, 36). In focal CHI, a paternally inherited faulty gene only manifests in a focal region of cells where loss of heterozygosity of the maternal allele occurs, but not in the rest of the pancreas. Thus K_ATP_ mutations identified in focal CHI are recessive. Since both inheritance of the paternal mutant allele and loss of the maternal allele are required for disease presentation, the recurrence among the siblings is rare; to our knowledge, it has only been reported once in two siblings (37). However, in consanguineous parents, mothers should be screened for the presence of the paternal mutation responsible for the focal form of CHI to avoid the possibility of diffuse CHI in future pregnancies due to inheritance of the mutation from both parents.

Unlike focal K_ATP_-HI, diffuse K_ATP_-HI may be dominant or recessive, which often correlates with whether the underlying mutation causes K_ATP_ channel gating or trafficking defects (see more discussion in “Mechanisms of K_ATP_-HI mutations”). Recessive mutations, whether homozygous or compound heterozygous, are usually found in patients with severe disease and not responsive to diazoxide treatment (38). Dominant mutations have been identified in patients with mild, diazoxide-responsive disease as well as severe, diazoxide-unresponsive disease (39–43). For genetic counseling, homozygous recessive mutations are expected to have a recurrence rate of ~25%, while dominant heterozygous mutations have a recurrence rate up to 50%. Differentiating between dominant and recessive mutations can in some cases be challenging. For example, penetrance of a mutation may not be the same in all carriers (44). Differential expression of a dominant *ABCC8* mutation has been observed in lymphocytes from two different carriers and proposed to account for the difference in their clinical presentation of CHI (45). Moreover, the pedigrees of CHI patients are often too small to clarify the inheritance pattern of novel mutations. Combining clinical, genetic, and functional studies using *in vitro* recombination systems can help resolve ambiguous cases.

## Mechanisms of K_ATP_-HI mutations

**Figure 2:**
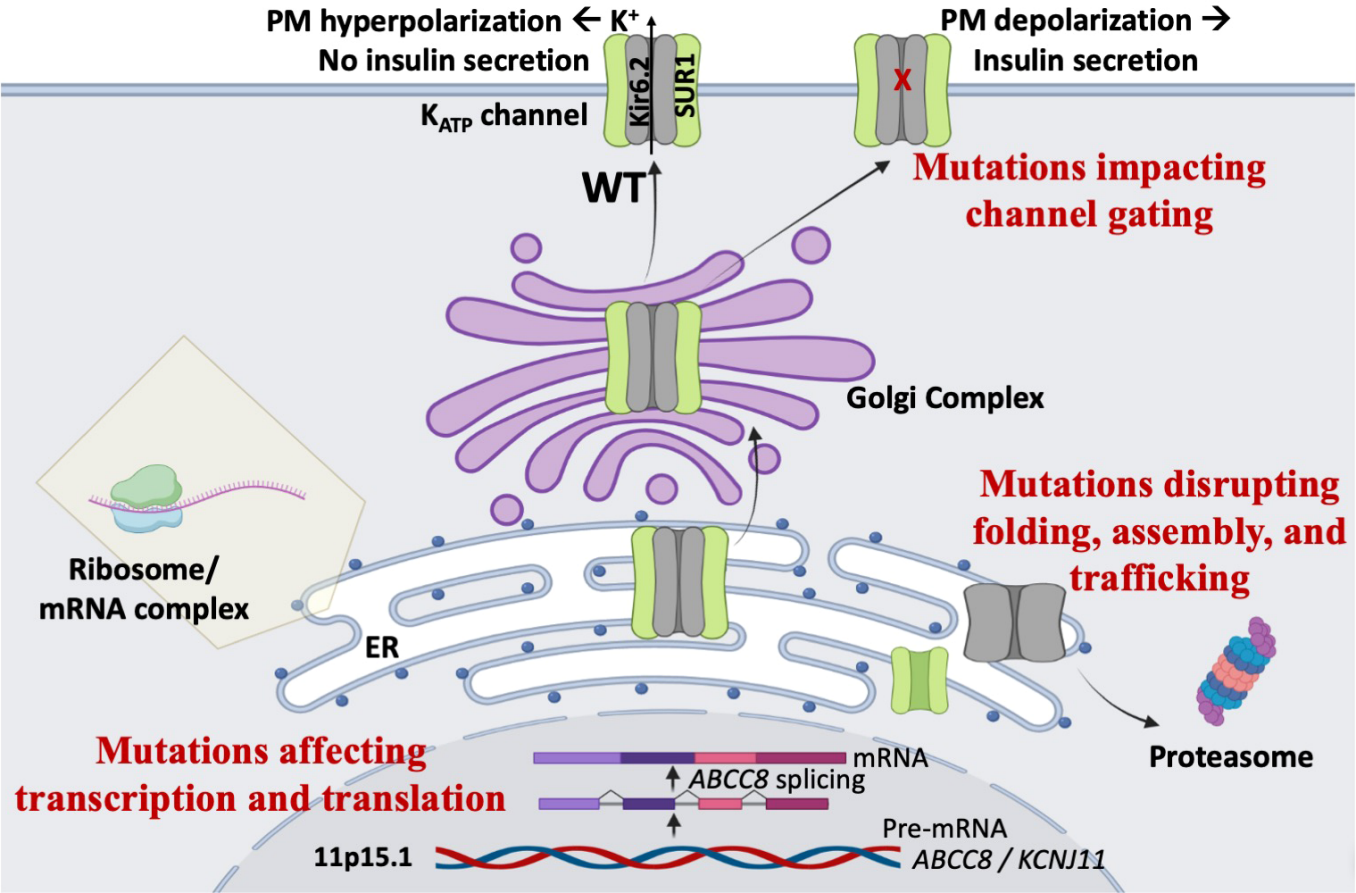
Mechanisms of K_ATP_-HI mutations. Mutations in the K_ATP_ channel genes can lead to loss of channel function, persistent plasma membrane (PM) depolarization, and inappropriate insulin secretion *via* multiple mechanisms. First, mutations may impair gene transcription or protein translation. Second, mutations may disrupt K_ATP_ channel protein folding, assembly, and trafficking, thereby compromising surface expression of the channel. Third, mutations may cause gating defects that prevent channel opening when blood glucose levels decline.

*ABCC8* contains 42 exons encoding 1581 amino acids (or a common alternative isoform with 1582 residues), whereas *KCNJ11* contains a single exon encoding 390 amino acids. Genetic variants in *ABCC8* and *KCNJ11* are found in both non-coding and coding regions. We have divided them into three broad classes: those causing impairment of transcription and translation, those disrupting folding, assembly, and trafficking, and those disrupting gating of K_ATP_ channels (**Figure 2**).

In non-coding regions, genetic variations can in principle affect gene expression, or splicing in the case of *ABCC8* to reduce transcript copies, and thereby K_ATP_ expression and function. Few studies have examined regulation of *ABCC8* and *KCNJ11* genes in pancreatic β-cells by cis-elements in the non-coding regions (46, 47). Definitive demonstration that a variation in these regions reduce transcript number would be very difficult. Several studies have presented *in vitro* evidence that genetic variations could disrupt splicing of SUR1 transcripts, especially variants located near the *ABCC8* intron-exon boundaries, using a combination of bioinformatics and expression of mini-genes containing the variants or digital droplet PCR of patient lymphocytes (31, 48–51).

Mutations in the coding regions can introduce premature stop codons or frameshift, resulting in truncated, nonfunctional proteins, but can also be silent, i.e. without changing the encoded amino acid. Silent mutations could potentially affect protein translation and folding by altering mRNA structure or codon usage (52); however, this possibility has not been tested. Most commonly, mutations in the coding regions alter primary sequence of SUR1 or Kir6.2. These include missense mutations and indel mutations. Alterations in the primary sequence of channel proteins can reduce or abolish channel function by disrupting channel folding, assembly, trafficking and/or gating response to blood glucose levels (26, 27, 53).

Understanding how novel K_ATP_ channel missense/indel mutations affect channel expression and function greatly facilitates molecular diagnosis and therapeutic management of CHI. Currently, *in silico* methods are unable to accurately predict the functional impact of a mutation, and native β-cells from patients are mostly unavailable. Endocrinologists around the world collaborate with several academic laboratories including ours to characterize effects of these mutations in recombinant expression systems using biochemical and electrophysiological assays (54). For these studies, recombinant mutant channels are transiently expressed in a mammalian cell line that does not express endogenous K_ATP_ channels, such as COSm6 or HEK293 cells. To assess the impact of a mutation on channel properties, mutant channels are first expressed and evaluated as a homogeneous population mimicking homozygous state. For heterozygous mutations, follow-up studies where the mutant is co-expressed with the wild-type at 1:1 ratio to simulate the heterozygous state may be performed to determine whether a mutation has a dominant effect over the wild-type allele on channel expression and function (30).

A rapid and informative method to determine whether a mutation is pathogenic is the Rb^+^ efflux assay (54, 55). In this assay, Rb^+^, which passes through K_ATP_ channels, is a tracer ion that acts as a surrogate K^+^ and can be detected using a radioactive form of Rb^+^, ^86^Rb^+^ (54), or by atomic absorption spectroscopy (56), to monitor K_ATP_ channel activity. In β-cells, K_ATP_ channels open in response to glucose deprivation, which lowers the intracellular ATP/ADP ratio. In COSm6 cells, which are not glucose-responsive, reduction of ATP/ADP ratios can be triggered by incubating cells with metabolic inhibitors including the glycolysis inhibitor 2-deoxyglucose and the oxidative phosphorylation inhibitor oligomycin, which reduce ATP production. In COSm6 cells transiently expressing K_ATP_ channels and preloaded with Rb^+^, opening of K_ATP_ channels leads to increased Rb^+^ efflux. Reduced efflux observed in cells expressing channels harboring a mutation compared to cells expressing wild-type channels would indicate that the mutation causes loss of channel function, therefore has a pathogenic role in CHI. In addition to its utility in evaluating the pathogenic role of a mutation, the Rb^+^ efflux assay is also useful for assessing whether a mutant form of the channel functionally responds to the K_ATP_ channel opener diazoxide, a frontline treatment for CHI (57). Restoration of Rb^+^ efflux by diazoxide would indicate a clinical response of patients with the mutation to diazoxide treatment. Indeed, there is in general good agreement between response in Rb^+^ efflux assays and clinical response to diazoxide based on published work (29, 40, 42, 58). However, phenotypical variations caused by the same mutation could result in patient response that deviates from prediction based on Rb^+^ efflux assays using recombinant mutant channels expressed in cultured cells.

Mutations which reduce K_ATP_ channel activity in Rb^+^ efflux assays can disrupt the ability of the channel to open at low glucose concentrations (gating defects) and/or reduce the number of channels present in the plasma membrane (trafficking defects). The precise mechanisms can be further determined using biochemical and electrophysiological assays, which will aid in the decision on disease treatment plans.

### Mutations disrupting channel gating

**Figure 3:**
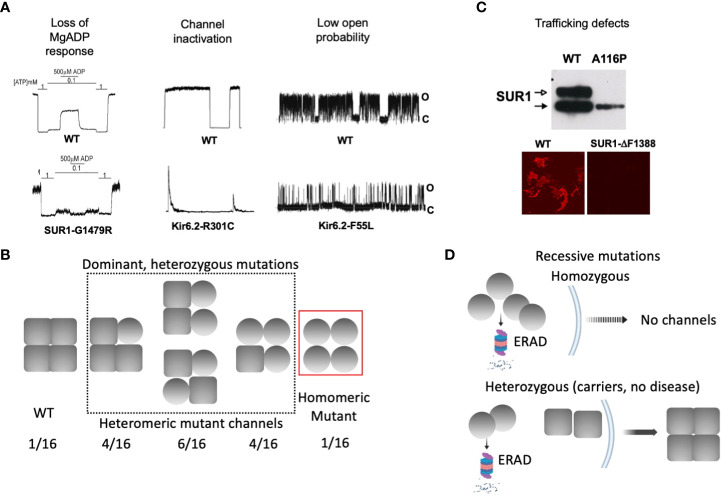
Biochemical and functional characteristics of K_ATP_ channel mutations associated with dominant versus recessive diffuse CHI. **(A)** Gating defects that have been observed in CHI-associated mutations, including loss of MgADP response, spontaneous current decay. i.e. channel inactivation, and reduced channel open probability. For each defect, representative inside-out patch-clamp recordings of WT and homomeric mutant channels are shown. For monitoring MgADP response, channels were exposed to 1 mM ATP, 0.1 mM ATP, or 0.1 mM ATP plus 0.5 mM MgADP as indicated. In all recordings, channel openings (O) are shown as upward deflections, and channel closures **(C)** as flat baselines. **(B)** Schematic showing that gating mutations tend to be associated with dominant disease, where heterozygous patients are expected to express a mixed population of channels containing 0-4 mutant subunits. In these cases, the mutation causes gating defects but not defects in channel folding, assembly, and trafficking. **(C)** *Top*: A western blot showing both core-glycosylated and complex-glycosylated wild-type SUR1 band when co-expressed with Kir6.2, indicating channel assembly and ability to traffic to the cell surface. In contrast, a trafficking mutation SUR1-A116P, fails to generate the mature complex-glycosylated band. *Bottom*: Surface immunofluorescence staining showing lack of expression of a trafficking mutant SUR1-ΔF1388, in contrast to WT. **(D)** Schematic showing trafficking mutations are usually seen in recessive disease. In patients carrying homozygous mutations, mutant protein is targeted for ER-associated proteasomal degradation (ERAD) and is unable to form channels and traffic to the plasma membrane. In heterozygous individuals, mutant protein is degraded and unable to assemble with WT subunit, leaving WT protein to assemble into functional channels that traffic to the plasma membrane, escaping the disease.

Accurate response of K_ATP_ channels to changes in intracellular ATP and MgADP concentrations is essential for glucose-insulin secretion coupling (25). In addition, channel activity relies on interactions with membrane phospholipids, in particular PI4,5P_2_ (PIP_2_) (59, 60). Patch-clamp recordings of K_ATP_ channels using the inside-out configuration allows for precise control of the solution on the intracellular face of channels contained in a membrane patch to evaluate channel response to the above physiological ligands. The most common gating defect seen in CHI-associated mutations is impaired response to MgADP/MgATP (61, 62) (**Figure 3A**), which stimulates K_ATP_ channels by binding to the SUR1 nucleotide binding domains (NBDs). Accordingly, these mutations are almost exclusively located in SUR1, and many in the NBDs (62). In general, disease severity correlates with the extent of nucleotide response impairment (30, 40, 42, 58). Moreover, mutations that impair MgADP response also tend to impair channel response to diazoxide (40, 58), which acts by stabilizing SUR1 in MgADP/MgATP stimulated conformation (23). As diazoxide is the only K_ATP_-targeting drug currently available to treat CHI, patients with mutations that cause severe impairment of MgADP response often fail to respond to diazoxide and require alternative interventions (63). Less common are mutations which reduce the open probability of the channel, for example Kir6.2 mutations that reduce channel response to membrane PIP_2_ (64) or render the current unstable by disrupting Kir6.2 subunit-subunit interactions (65) (**Figure 3A**). Channels with these mutations generally remain responsive to MgADP and diazoxide (64, 65).

In diffuse K_ATP_-HI, gating mutations often follow a dominant inheritance pattern (**Figure 3B**). Because each K_ATP_ channel contains four Kir6.2 and four SUR1 subunits, heterozygous mutations presumably generate a mixed channel population containing 0, 1, 2, 3, or 4 mutant subunits at a ratio of 1:4:6:4:1, with the gating defect more pronounced as the number of mutant subunit increases. A subunit with a mutation that affects channel gating, but that is able to co-assemble and traffic to the cell surface, would have its gating defect manifested in the total surface channel population. The extent of the MgADP and diazoxide gating defect has been correlated with disease severity and clinical response to diazoxide in CHI children with dominant SUR1 mutations (40, 58). Thus, response of mutant channels to MgADP and diazoxide in electrophysiology experiments may be helpful in predicting disease phenotype and clinical response to diazoxide.

### Mutations disrupting channel folding, assembly and trafficking

Many CHI mutations cause improper channel folding, assembly, and trafficking to the plasma membrane (53). The consequent reduction in K_ATP_ currents leads to a state of persistent β-cell membrane depolarization and uncontrolled insulin secretion. Translation, folding, and assembly of K_ATP_ channel subunits occur in the endoplasmic reticulum (ER) membrane. Upon correct assembly into a hetero-octameric complex, channels exit the ER and traffic to the Golgi. In the Golgi apparatus, SUR1 becomes complex glycosylated at its two N-linked glycosylation sites, giving rise to a mature form that migrates slower on SDS gel compared to the core-glycosylated immature form found in the ER (66, 67). The appearance and intensity of the mature SUR1 band can be used to infer channel proteins that are competent to traffic to the plasma membrane. Conversely, the absence or weakened mature SUR1 band indicates folding/assembly/trafficking defects (**Figure 3C**). More direct assessment of K_ATP_ channel surface expression can be achieved by surface immunofluorescence staining, surface biotinylation followed by pulldown of biotinylated protein and immunoblotting with anti-SUR1 antibody, or by electrophysiological measurement of current density (68–71).

**Figure 4:**
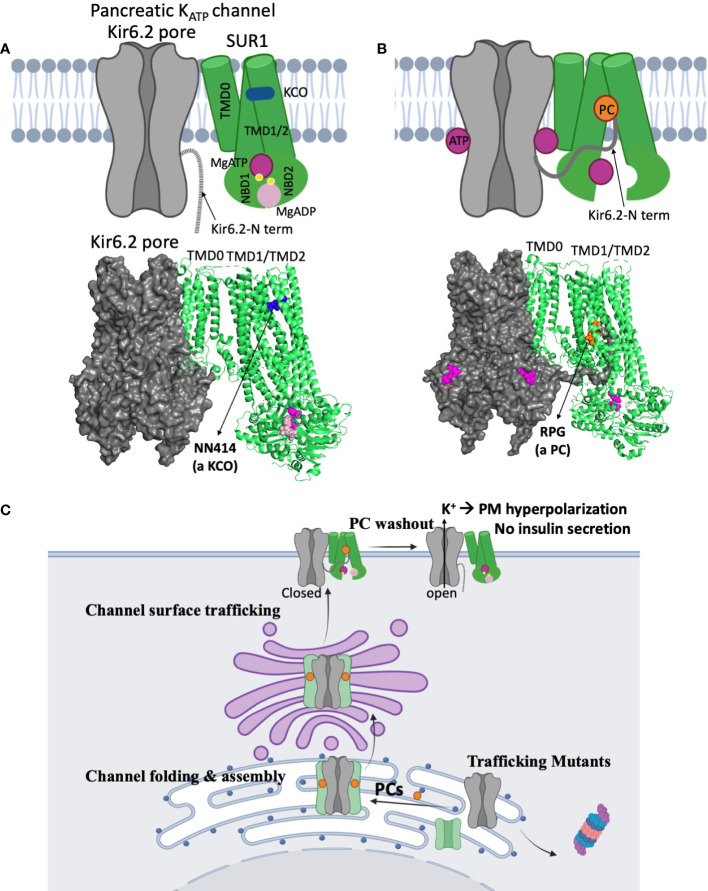
Structural insights on the mechanisms of K_ATP_ channel pharmacological modulators. **(A)** A cartoon model (*top*) illustrating the mechanism by which potassium channel openers (KCOs) stimulate channel activity, based on a recent structure of a channel bound to MgATP/MgADP and a KCO NN414 shown below (PDB ID: 7W4O; only one SUR1 subunit shown for clarity). In the structure, the SUR1 NBD1 and NBD2 are bound to MgATP and MgADP respectively and dimerized, a conformation that stimulates channel activity. NN414, which binds at a transmembrane domain pocket further stabilizes channels in the SUR1-NBDs dimerized, activated conformation to potentiate channel opening. By inference, diazoxide, a KCO used to treat some CHI patients, promotes channel opening *via* a similar mechanism. **(B)** A cartoon (*top*) showing how K_ATP_ channel inhibitors work as pharmacological chaperones (PC) based on cryoEM structures. A channel structure determined in the presence of ATP and a PC repaglinide is shown at the bottom (PDB ID: 7U1S). The structure shows that the PC (orange circle), binds in a transmembrane pocket in SUR1 formed by helices from TMD1 and TMD2. The Kir6.2 N-terminus is in a cavity formed by the two transmembrane helix bundles (TMD1/2) above the two NBDs, and is adjacent to the bound PC. This stabilizes the interaction between the N-terminal domain of Kir6.2 and SUR1 to facilitate the formation of mature hetero-octameric complex of the mutant channel. However, in this conformation the NBDs of SUR1 are separate, unable to respond to MgADP stimulation upon glucose deprivation. This explains why K_ATP_ PCs also inhibit channel activity. **(C)** A cartoon showing a proposed mechanism of how reversible inhibitor PCs can enhance channel surface expression without permanently compromising channel function. Inhibitor PCs bind to the mutant channel subunits and enhance the interaction between SUR1 central cavity and Kir6.2 N-terminus to promote mutant channel assembly and surface trafficking. Reversible inhibitor PCs are released upon washout after mutant channels are rescued to the surface, allowing the channels to open under low blood glucose conditions and inhibit insulin secretion.

To date, about fifty missense/indel SUR1 and Kir6.2 mutations have been reported to impair K_ATP_ channel expression at the cell surface, collectively referred to as trafficking mutations (29, 30, 53, 70, 72, 73). The extent of the impairment varies, with some mutations completely abolishing the mature SUR1 band and others reducing but not eliminating mature SUR1 (71, 74). A prominent example of the former is SUR1ΔF1388 (68), a common recessive mutation found in the Ashkenazi Jewish population (75). The mutant protein is retained in the ER, unable to reach the mature complex-glycosylated state likely because it is misfolded (68), akin to the most prevalent cystic fibrosis-causing mutation CFTRΔF508 (76). Although trafficking mutations are mapped throughout the SUR1 and Kir6.2 proteins, a high percentage are found in the first transmembrane domain of SUR1, called TMD0, and the first transmembrane helix (TM1) of Kir6.2 (53, 72). Recent high resolution 3D structures of the channel complex show that SUR1-TMD0 makes direct contact with Kir6.2-TM1, forming the primary anchor between the two subunits (77–79) (**Figure 4**). Mutations in these domains likely interfere with channel assembly, and thereby the trafficking of channels to the plasma membrane.

In contrast to gating mutations, trafficking mutations reported to date have been associated with recessive CHI and do not respond to diazoxide treatment (15). The recessive nature of these mutations implies that under heterozygous condition the mutant allele may be too defective or is outcompeted by the wild-type allele to form a channel complex. Indeed, some trafficking mutations have been shown to reduce subunit association, and some have been shown to cause rapid degradation of channel proteins (72, 80). This leaves the wild-type subunit to assemble and form normal functional channels, resulting in a haplosufficiency phenotype (**Figure 3D**). It is possible that certain mutations that cause only mild trafficking defects such that mutant proteins are still able to assemble with the wild-type allele and reach the cell surface, can remain undetected in heterozygous conditions where the mutation does not disrupt gating sufficiently to cause disease. In this regard, it is interesting to note that many heterozygous K_ATP_ mutations identified in neonatal diabetes have been shown to cause trafficking defects in addition to gain-of-function gating defects. These mutant proteins are still able to form complex with wild-type subunit and exert their gain-of-function gating effect to cause dominant disease (81–83).

## Treatment of K_ATP_-HI: Challenges and opportunities

Early diagnosis and effective treatment are critical to prevent serious neurocognitive impairments that dramatically impact CHI patients and their families due to severe associated morbidity with lifelong disability (3, 57). For focal K_ATP_-HI, complete surgical removal of the lesion often leads to a cure (33). However, treatment for diffuse K_ATP_-HI remains challenging. Current mainstay treatments include diazoxide, somatostatin analogues, continuous enteral feedings/dextrose, and surgery (3, 57). Diazoxide treatment would be preferred if patients have β-cell K_ATP_ channels that have sufficient response to the drug. However, many patients do not respond to diazoxide. Even with patients who do respond to diazoxide, there is often significant side effects (3, 11, 84). Currently, diazoxide is the only K_ATP_ channel opener approved in the US and Europe. Diazoxide not only activates pancreatic K_ATP_ channels but also vascular K_ATP_ channels, causing excess hair growth and other cardiovascular complications resembling Cantú syndrome patients carrying gain of function mutations in vascular K_ATP_ channels (85). Continuous feeding/dextrose infusion can restore normal glycemia, but is a heavy burden on caretakers and patients and is associated with many complications including rapid weight gain and food aversion (3). For patients with diffuse disease and unresponsive to diazoxide and somatostatin analogues, pancreatectomy is often performed to correct the life-threatening hypoglycemia. This leads to insulin dependency later in life and digestive complications from removal of exocrine tissues (86).

With better understanding of the molecular mechanism underlying insulin secretion regulation and CHI, off label use of other drugs, such as somatostatin analogues including longer-acting octreotide (sandostatin LP, monthly injections rather than daily), glucagon, GLP-1 receptor antagonists (exendin 9-39), mTOR inhibitors (sirolimus), the calcium channel blocker nifedipine, and anti-insulin receptor antibody have been considered for CHI patients who are unresponsive to the maximum dose of diazoxide (87). However, these alternative treatments have limited success or are still in clinical trials, and do not target the root causes of K_ATP_ dysfunction in diazoxide-unresponsive diffuse CHI, namely, severe MgADP/diazoxide response defects or impaired channel trafficking to the plasma membrane. Overcoming these limitations requires better understanding of the structural mechanisms underlying channel function, dysfunction, and pharmacology.

### K_ATP_ channel structures and implications for K_ATP_-HI

A major advance in K_ATP_ channel research in recent years is our ability to visualize 3D channel structures at near atomic resolution. Using single-particle cryogenic electron microscopy (cryoEM), structures of pancreatic K_ATP_ bound to various inhibitors and activators have been determined (53, 56, 77–79, 88–91). These structures not only reveal the binding sites of key physiological and pharmacological ligands of K_ATP_ channels but also provide insights to the mechanisms of how ligands regulate channel assembly and function (17). The knowledge is of great value in future efforts to expand K_ATP_ channel pharmacology and overcome current therapeutic challenges.

Of particular interest are recent studies showing the pancreatic, cardiac, and vascular K_ATP_ channel SUR subunits, SUR1, SUR2A, and SUR2B, respectively, bound to their selective activators, NN414 (for SUR1) and P1075 or levcromakalim (for SUR2A and SUR2B) (56, 92). In the structure of SUR1 bound to NN414, NN414 sits in a transmembrane pocket that is stabilized by dimerization of the MgADP/MgATP bound NBDs of SUR1 (**Figure 4A**). A similar binding location for P1075 or levcromakalim is observed in SUR2A/2B. Although no diazoxide bound structure is yet available, diazoxide most likely binds to the same pocket. The structures help to explain why the effect of diazoxide requires MgADP/MgATP and why mutations that disrupt MgADP response also impair diazoxide response. Of note, NN414 stimulates K_ATP_ channel activity like diazoxide, but is more potent and selective for SUR1 containing pancreatic K_ATP_ channels (93, 94). NN414 was previously tested in clinical trials for type 2 diabetes based on the idea that opening of pancreatic K_ATP_ will allow β-cell rest and restore insulin secretion; however, the trial was stopped due to concerns over elevated liver enzymes (94). Whether NN414 can be used to treat CHI at concentrations that do not elicit hepatic or Cantú-like side effects remains untested. Regardless, the structures of different SUR isoforms bound to different openers (17, 19) provide a framework for rational drug design with improved specificity for pancreatic K_ATP_ channels without undesirable side effects to expand the medical options for CHI.

A significant number of K_ATP_ gating mutations result in channels with little or no MgADP and diazoxide response (30, 40, 58). For these mutations, drugs that open channels independent of the stimulatory effect of MgADP on SUR1 may be explored. For example, recent channel structures showing an open Kir6.2 pore conformation (56, 89) could be used to search for compounds, *via* virtual screening, that may stabilize the channel in an open state.

Lastly, mutations which impair channel expression at the cell surface also require novel approaches. Here, pharmacological chaperones, small molecules that bind to K_ATP_ channel proteins to facilitate biogenesis, correct misfolding, and restore full or partial function of the affected channels represents a promising approach (53, 95). This approach has been highly successful in cystic fibrosis, a disease caused by mutations in the CFTR gene. CFTR is a chloride channel that shares structural similarity with SUR1 (96), the regulatory subunit of K_ATP_ channels. The most common cystic fibrosis-causing mutation is ΔF508, which impairs folding, and thereby surface expression of the protein (76). Several small molecules that correct the folding and trafficking defect of mutant CFTR (lumacaftor, tezacaftor, and elexacaftor) have been recently approved for clinical use (76, 97, 98).

Sulfonylureas were the first reported K_ATP_ pharmacological chaperones (99). These antidiabetics, which inhibit K_ATP_ channels and stimulate insulin secretion, include the high affinity sulfonylurea glibenclamide and the low affinity sulfonylurea tolbutamide. Subsequently, glinides, a second class of K_ATP_ inhibiting antidiabetics including repaglinide, was found to have similar pharmacological chaperone effects (100). More recently, carbamazepine, an anticonvulsant known to block voltage-gated sodium channels was also discovered as a K_ATP_ pharmacological chaperone (69). Interestingly, these compounds are all K_ATP_ channel inhibitors (69, 101). Moreover, each of these K_ATP_ channel inhibitors are effective for only a subset of trafficking mutations. In particular, they promote trafficking of channels harboring mutations in the TMD0 domain of SUR1, which is the primary domain that interacts with Kir6.2 (69, 100), suggesting drug binding corrects trafficking defects by facilitating subunit assembly (102). By examining cryoEM structures of channels bound to glibenclamide, repaglinide, or carbamazepine, it was discovered that the distal N-terminus of Kir6.2 cooperates with SUR1 for drug binding, and drug binding in turn stabilizes Kir6.2-N terminus interaction with SUR1 (103) (**Figure 4B**). Thus, these drugs likely exert their channel chaperoning and inhibition effects *via* the same mechanism (**Figure 4B**), i.e. by stabilizing the Kir6.2 N-terminus in the SUR1 ABC core cavity. By contrast, channel openers such as diazoxide and NN414, which stabilize SUR1-NBD dimerization and exclude Kir6.2 N-terminus from the SUR1 ABC core cavity (**Figure 4A**), diminish Kir6.2-SUR1 interactions and have been shown to lack chaperoning activity (74).

While promising, translation of the above basic science finding to CHI treatment has a number of challenges. First, all K_ATP_ pharmacological chaperones reported to date are inhibitors. The most potent chaperones like glibenclamide and repaglinide are also the most potent inhibitors. The nearly irreversible inhibition of channels by these high affinity inhibitors precludes functional recovery of mutant channels rescued to the cell surface (99). To date, tolbutamide is by far the most reversible inhibitor that is also effective in rescuing mutant channels to the cell surface, albeit at significantly higher concentrations (99). Tolbutamide, a first-generation sulfonylurea which has been around since the 1950s, has largely been replaced with other oral hypoglycemics and is no longer available in the US. While early randomized trials associated its use with increased cardiovascular and all-cause mortality, the increased cardiovascular and all-cause mortality was not statistically significant (104). Off label use of tolbutamide as a potential pharmacologic therapy for CHI patients with diffuse disease and K_ATP_ trafficking mutations is an intriguing possibility (**Figure 4C**). Secondly, some trafficking mutations also disrupt channel gating. For example, two CHI-causing SUR1 trafficking mutations, R74W and E128K, also render channels less sensitive to ATP inhibition such that upon pharmacological rescue to the plasma membrane mutant channels cause membrane hyperpolarization and decreased glucose-stimulated insulin secretion in cultured insulinoma cells that resemble neonatal diabetes mutation phenotypes (105). Thirdly, not all trafficking mutations respond to the K_ATP_ pharmacological chaperones identified to date (71, 99). These mutations, largely located outside TMD0 of SUR1, likely cause severe misfolding such that mutant proteins are triaged for degradation (99, 106). Additional drug screening studies such as those done for CFTRΔF508 will be required to overcome defects caused by such mutations. Finally, there is currently no animal models to test the *in vivo* feasibility of pharmacological chaperone therapy. Recent development of induced pluripotent stem cells (iPSCs) derived from a CHI patient carrying a homozygous trafficking mutation SUR1-V187D (107) could serve as an intermediate experimental model to test the effect of reversible K_ATP_ pharmacological chaperones such as tolbutamide.

## Concluding remarks

Much progress has been made in the diagnosis and management of K_ATP_-HI since the first report linking K_ATP_ channel gene mutations to CHI (108). There are now hundreds of mutations that have been identified, and there has been steady progress in our understanding of genotype-phenotype correlation, mutation mechanisms, and drug response. The growing knowledge base facilitates rapid diagnosis and treatment. Despite the progress, timely and accurate molecular diagnosis of patients carrying variants of unknown significance, and treatment of diazoxide-unresponsive diffuse disease caused by severe gating and trafficking mutations remain challenging. With recent rapid technical advances in gene sequencing, bioinformatics, channel structure determination, machine-learning based drug design, and gene therapy, there is great optimism that new and personalized therapies for K_ATP_-HI will become a reality in the not-too-distant future.

## Author contributions

All authors contributed to the article and approved the submitted version.
